# Crystal structure of pymetrozine

**DOI:** 10.1107/S2056989015010804

**Published:** 2015-06-10

**Authors:** Youngeun Jeon, Jineun Kim, Gihaeng Kang, Tae Ho Kim

**Affiliations:** a Department of Chemistry and Research Institute of Natural Sciences, Gyeongsang National University, Jinju 660-701, Republic of Korea

**Keywords:** crystal structure, pymetrozine, triazinone, insecticide, anti­feedant, hydrogen bonding, π–π inter­actions

## Abstract

The title compound, C_10_H_11_N_5_O {systematic name: 6-methyl-4-[(*E*)-(pyridin-3-yl­methyl­idene)amino]-4,5-di­hydro-1,2,4-triazin-3(2*H*)-one}, C_10_H_11_N_5_O, is used as an anti­feedant in pest control. The asymmetric unit comprises two independent mol­ecules, *A* and *B*, in which the dihedral angles between the pyridinyl and triazinyl ring planes [r.m.s. deviations = 0.0132 and 0.0255 ] are 11.60 (6) and 18.06 (4)°, respectively. In the crystal, N—H⋯O, N—H⋯N, C—H⋯N and C—H⋯O hydrogen bonds, together with weak π–π inter­actions [ring-centroid separations = 3.5456 (9) and 3.9142 (9) Å], link the pyridinyl and triazinyl rings of *A* mol­ecules, generating a three-dimensional network.

## Related literature   

For information on the toxicity and insecticidal properties of the title compound, see: He *et al.* (2011 ▸); Torres *et al.* (2003 ▸); Ausborn *et al.* (2005 ▸); Barati *et al.* (2013 ▸). For a related crystal structure, see: Wang *et al.* (2012 ▸).
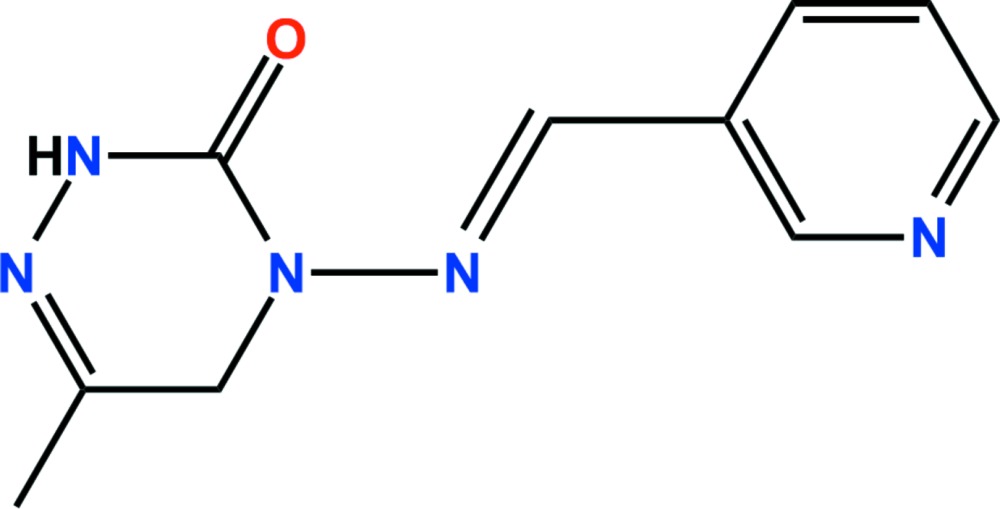


## Experimental   

### Crystal data  

C_10_H_11_N_5_O
*M* *_r_* = 217.24
Monoclinic, 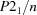

*a* = 8.0803 (2) Å
*b* = 23.7497 (6) Å
*c* = 10.7846 (3) Å
β = 98.962 (1)°
*V* = 2044.35 (9) Å^3^
*Z* = 8
Mo *K*α radiation
μ = 0.10 mm^−1^
*T* = 173 K
0.37 × 0.16 × 0.09 mm

### Data collection  

Bruker APEXII CCD diffractometer
Absorption correction: multi-scan (*SADABS*; Bruker, 2009 ▸) *T*_min_ = 0.964, *T*_max_ = 0.991
19037 measured reflections
3987 independent reflections
3103 reflections with *I* > 2σ(*I*)
*R*_int_ = 0.042

### Refinement  

*R*[*F* ^2^ > 2σ(*F* ^2^)] = 0.041
*wR*(*F* ^2^) = 0.107
*S* = 1.04
3987 reflections
291 parameters
H-atom parameters constrained
Δρ_max_ = 0.19 e Å^−3^
Δρ_min_ = −0.31 e Å^−3^

### 

Data collection: *APEX2* (Bruker, 2009 ▸); cell refinement: *SAINT* (Bruker, 2009 ▸); data reduction: *SAINT*; program(s) used to solve structure: *SHELXTL* (Sheldrick, 2008 ▸); program(s) used to refine structure: *SHELXTL*; molecular graphics: *DIAMOND* (Brandenburg, 2010 ▸); software used to prepare material for publication: *SHELXTL*.

## Supplementary Material

Crystal structure: contains datablock(s) global, I. DOI: 10.1107/S2056989015010804/sj5463sup1.cif

Structure factors: contains datablock(s) I. DOI: 10.1107/S2056989015010804/sj5463Isup2.hkl

Supporting information file. DOI: 10.1107/S2056989015010804/sj5463Isup3.cml

**Fig. 1.**
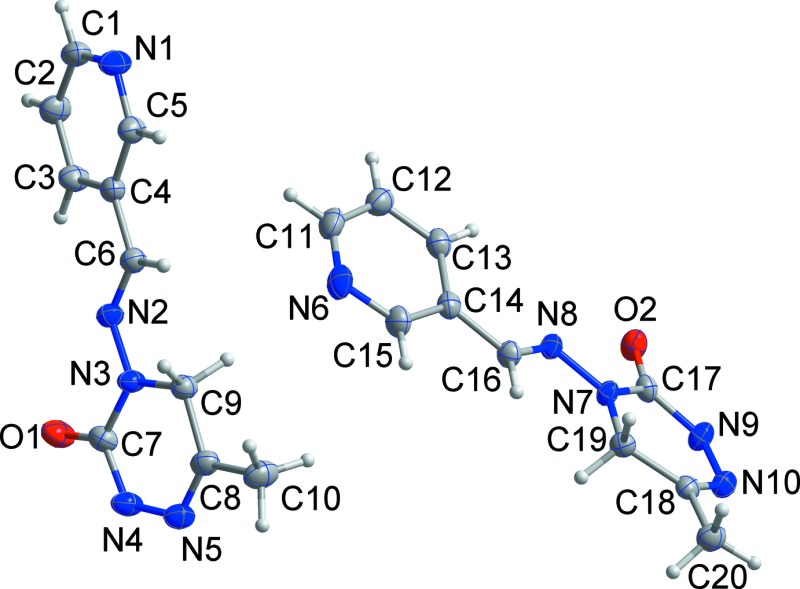
The asymmetric unit of the title compound with the atom-numbering scheme. Displacement ellipsoids are drawn at the 50% probability level. H atoms are shown as small spheres of arbitrary radius.

**Fig. 2.**
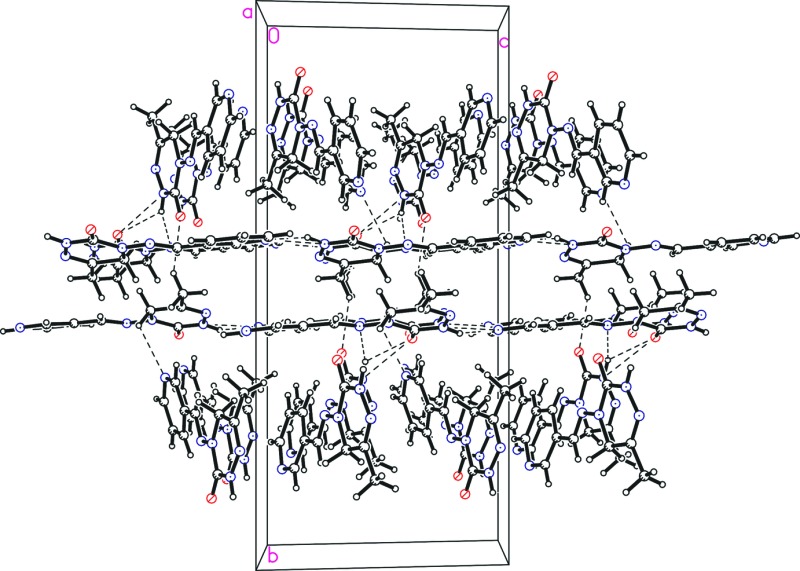
Crystal packing viewed along the a axis. The hydrogen bonds are shown as dashed lines.

. DOI: 10.1107/S2056989015010804/sj5463fig1.tif
The asymmetric unit of the title compound with the atom-numbering scheme. Displacement ellipsoids are drawn at the 50% probability level. H atoms are shown as small spheres of arbitrary radius.

a . DOI: 10.1107/S2056989015010804/sj5463fig2.tif
Crystal packing viewed along the *a* axis. The hydrogen bonds are shown as dashed lines.

CCDC reference: 1404941

Additional supporting information:  crystallographic information; 3D view; checkCIF report

## Figures and Tables

**Table 1:** Hydrogen-bond geometry (, )

*D*H*A*	*D*H	H*A*	*D* *A*	*D*H*A*
N4H4O2^i^	0.88	2.32	2.9545(17)	129
N4H4N7^i^	0.88	2.43	3.2346(17)	152
N9H9N6^ii^	0.88	2.04	2.882(2)	159
C19H19*A*N1^iii^	0.99	2.57	3.0852(19)	112
C19H19*A*O1^iv^	0.99	2.60	3.5596(19)	164
C20H20*C*O1^v^	0.98	2.53	3.3906(19)	147

Symmetry codes: (i) 
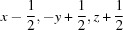
; (ii) 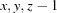; (iii) 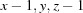; (iv) 
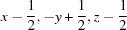
; (v) 
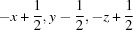
.

## supplementary crystallographic information

### S1. Comment

Pymetrozine: 4,5-dihydro-6-methyl-4-[(*E*)-(3-pyridinylmethylene)amino]-1,2,4-triazin-3(2*H*)-one, is an insecticide for the control of sucking insects, including the brown planthopper, Nilaparvata lugens, one of the most serious pests to affect rice crops (He *et al.*, 2011). Its crystal structure is reported herein. In the title compound (Fig. 1), the asymmetric unit comprises two independent molecules (A and B) and the dihedral angles between the pyridinyl and triazinyl ring planes are 11.60 (6) and 18.06 (4)° for A and B, respectively. All bond lengths and bond angles are normal and comparable to those observed in a similar crystal structure (Wang *et al.*, 2012).

The crystal structure, Fig. 2, is stabilized by intermolecular N—H···O, N—H···N, C—H···N and C—H···O hydrogen bonds (Table 1). In addition, weak intermolecular π···π interactions between the pyridinyl and triazinyl rings link adjacent A molecules [*Cg*1···*Cg*2^i^, 3.5456 (9) Å and *Cg*1···*Cg*2^ii^, 3.9142 (9) Å] symmetry codes: (i) = *x* + 1/2, -*y* + 1/2, *z* + 1/2, (ii) = *x* + 1, *y*, *z*; *Cg*1 and *Cg*2 are the centroids of the N1···C5 and N3···C9 rings, respectively]. The π···π interactions together with C1–H1···N5 hydrogen bonds generate sheets of A molecules in the *ac* plane. All of these contacts combine to generate a three dimensional network, Fig. 2.

### S2. Experimental

The title compound was purchased from the Dr. Ehrenstorfer GmbH Company. Slow evaporation of a solution in CH_2_Cl_2_ gave single crystals suitable for X-ray analysis.

### S3. Refinement

All H-atoms were positioned geometrically and refined using a riding model with d(N—H) = 0.88 Å, *U*_iso_ = 1.2*U*_eq_(C) for the amine group, d(C—H) = 0.98 Å, *U*_iso_ = 1.2*U*_eq_(C) for the methyl group, d(C—H) = 0.99 Å, *U*_iso_ = 1.2*U*_eq_(C) for methylene C—H and d(C—H) = 0.95 Å, *U*_iso_ = 1.2*U*_eq_(C) for aromatic H atoms

### Crystal data

**Table:**

C_10_H_11_N_5_O	*F*(000) = 912
*M**_r_* = 217.24	*D*_x_ = 1.412 Mg m^−^^3^
Monoclinic, *P*2_1_/*n*	Mo *K*α radiation, λ = 0.71073 Å
*a* = 8.0803 (2) Å	Cell parameters from 3981 reflections
*b* = 23.7497 (6) Å	θ = 2.7–27.3°
*c* = 10.7846 (3) Å	µ = 0.10 mm^−^^1^
β = 98.962 (1)°	*T* = 173 K
*V* = 2044.35 (9) Å^3^	Block, colourless
*Z* = 8	0.37 × 0.16 × 0.09 mm

### Data collection

**Table:**

Bruker APEXII CCD diffractometer	3987 independent reflections
Radiation source: fine-focus sealed tube	3103 reflections with *I* > 2σ(*I*)
Graphite monochromator	*R*_int_ = 0.042
φ and ω scans	θ_max_ = 26.0°, θ_min_ = 2.1°
Absorption correction: multi-scan (*SADABS*; Bruker, 2009)	*h* = −8→9
*T*_min_ = 0.964, *T*_max_ = 0.991	*k* = −28→29
19037 measured reflections	*l* = −13→12

### Refinement

**Table:**

Refinement on *F*^2^	Primary atom site location: structure-invariant direct methods
Least-squares matrix: full	Secondary atom site location: difference Fourier map
*R*[*F*^2^ > 2σ(*F*^2^)] = 0.041	Hydrogen site location: inferred from neighbouring sites
*wR*(*F*^2^) = 0.107	H-atom parameters constrained
*S* = 1.04	*w* = 1/[σ^2^(*F*_o_^2^) + (0.0528*P*)^2^ + 0.3325*P*] where *P* = (*F*_o_^2^ + 2*F*_c_^2^)/3
3987 reflections	(Δ/σ)_max_ = 0.001
291 parameters	Δρ_max_ = 0.19 e Å^−^^3^
0 restraints	Δρ_min_ = −0.31 e Å^−^^3^

### Special details

**Table:**

Geometry. All e.s.d.'s (except the e.s.d. in the dihedral angle between two l.s. planes) are estimated using the full covariance matrix. The cell e.s.d.'s are taken into account individually in the estimation of e.s.d.'s in distances, angles and torsion angles; correlations between e.s.d.'s in cell parameters are only used when they are defined by crystal symmetry. An approximate (isotropic) treatment of cell e.s.d.'s is used for estimating e.s.d.'s involving l.s. planes.
Refinement. Refinement of *F*^2^ against ALL reflections. The weighted *R*-factor *wR* and goodness of fit *S* are based on *F*^2^, conventional *R*-factors *R* are based on *F*, with *F* set to zero for negative *F*^2^. The threshold expression of *F*^2^ > σ(*F*^2^) is used only for calculating *R*-factors(gt) *etc*. and is not relevant to the choice of reflections for refinement. *R*-factors based on *F*^2^ are statistically about twice as large as those based on *F*, and *R*- factors based on ALL data will be even larger.

### Fractional atomic coordinates and isotropic or equivalent isotropic displacement parameters (Å^2^)

**Table:**

	*x*	*y*	*z*	*U*_iso_*/*U*_eq_	
O1	0.54588 (13)	0.37396 (4)	0.67454 (12)	0.0371 (3)	
O2	0.65704 (12)	0.09515 (4)	−0.11461 (10)	0.0298 (3)	
N1	1.18740 (16)	0.17122 (6)	0.90565 (15)	0.0353 (4)	
N2	0.70889 (14)	0.27719 (5)	0.72493 (13)	0.0269 (3)	
N3	0.54303 (15)	0.27730 (5)	0.67218 (13)	0.0272 (3)	
N4	0.31072 (15)	0.32889 (5)	0.58809 (14)	0.0333 (3)	
H4	0.2610	0.3618	0.5750	0.040*	
N5	0.21533 (15)	0.28219 (5)	0.54719 (14)	0.0314 (3)	
N6	0.56087 (18)	0.08007 (6)	0.54024 (13)	0.0362 (3)	
N7	0.53681 (14)	0.07233 (5)	0.09543 (12)	0.0226 (3)	
N8	0.43763 (14)	0.06556 (5)	−0.01909 (12)	0.0212 (3)	
N9	0.41093 (15)	0.07374 (5)	−0.23457 (12)	0.0273 (3)	
H9	0.4531	0.0850	−0.3008	0.033*	
N10	0.24931 (16)	0.05238 (5)	−0.25699 (13)	0.0266 (3)	
C1	1.28109 (19)	0.21759 (7)	0.91859 (17)	0.0325 (4)	
H1	1.3940	0.2145	0.9587	0.039*	
C2	1.22268 (19)	0.26979 (7)	0.87674 (17)	0.0346 (4)	
H2	1.2949	0.3016	0.8865	0.041*	
C3	1.05822 (19)	0.27537 (6)	0.82046 (17)	0.0319 (4)	
H3	1.0158	0.3109	0.7900	0.038*	
C4	0.95585 (18)	0.22816 (6)	0.80914 (15)	0.0253 (3)	
C5	1.02808 (19)	0.17746 (6)	0.85192 (16)	0.0305 (4)	
H5	0.9594	0.1448	0.8423	0.037*	
C6	0.77874 (18)	0.22966 (6)	0.75433 (16)	0.0269 (4)	
H6	0.7162	0.1958	0.7407	0.032*	
C7	0.47257 (18)	0.32978 (6)	0.64618 (16)	0.0275 (4)	
C8	0.28134 (18)	0.23414 (7)	0.57182 (16)	0.0281 (4)	
C9	0.45481 (18)	0.22477 (6)	0.63922 (16)	0.0274 (4)	
H9A	0.4495	0.2029	0.7167	0.033*	
H9B	0.5183	0.2022	0.5853	0.033*	
C10	0.1823 (2)	0.18265 (7)	0.53033 (19)	0.0397 (5)	
H10A	0.0639	0.1927	0.5073	0.060*	
H10B	0.1946	0.1552	0.5989	0.060*	
H10C	0.2234	0.1663	0.4575	0.060*	
C11	0.7273 (2)	0.08409 (7)	0.55839 (17)	0.0368 (4)	
H11	0.7839	0.0886	0.6418	0.044*	
C12	0.8219 (2)	0.08200 (7)	0.46212 (17)	0.0340 (4)	
H12	0.9406	0.0842	0.4797	0.041*	
C13	0.74117 (19)	0.07667 (6)	0.34017 (16)	0.0283 (4)	
H13	0.8032	0.0761	0.2722	0.034*	
C14	0.56786 (18)	0.07210 (6)	0.31837 (15)	0.0247 (3)	
C15	0.4844 (2)	0.07337 (7)	0.42216 (16)	0.0315 (4)	
H15	0.3661	0.0692	0.4080	0.038*	
C16	0.46794 (19)	0.06629 (6)	0.19390 (15)	0.0250 (3)	
H16	0.3517	0.0580	0.1860	0.030*	
C17	0.51141 (17)	0.07926 (6)	−0.12215 (14)	0.0219 (3)	
C18	0.18317 (18)	0.03964 (6)	−0.16097 (15)	0.0241 (3)	
C19	0.26507 (17)	0.04665 (6)	−0.02804 (15)	0.0233 (3)	
H19A	0.2010	0.0744	0.0140	0.028*	
H19B	0.2626	0.0103	0.0164	0.028*	
C20	0.01097 (18)	0.01533 (7)	−0.17992 (17)	0.0324 (4)	
H20A	−0.0309	0.0125	−0.2700	0.049*	
H20B	−0.0633	0.0397	−0.1402	0.049*	
H20C	0.0140	−0.0222	−0.1420	0.049*	

### Atomic displacement parameters (Å^2^)

**Table:**

	*U*^11^	*U*^22^	*U*^33^	*U*^12^	*U*^13^	*U*^23^
O1	0.0312 (6)	0.0260 (6)	0.0533 (9)	0.0021 (5)	0.0041 (5)	−0.0001 (6)
O2	0.0280 (6)	0.0374 (6)	0.0254 (7)	−0.0084 (4)	0.0079 (5)	−0.0027 (5)
N1	0.0292 (7)	0.0321 (7)	0.0420 (10)	0.0044 (6)	−0.0023 (6)	0.0034 (7)
N2	0.0211 (7)	0.0294 (7)	0.0295 (8)	0.0016 (5)	0.0015 (5)	0.0001 (6)
N3	0.0219 (6)	0.0254 (7)	0.0327 (8)	0.0026 (5)	−0.0004 (6)	0.0018 (6)
N4	0.0254 (7)	0.0264 (7)	0.0465 (10)	0.0062 (5)	0.0010 (6)	0.0034 (7)
N5	0.0243 (7)	0.0336 (7)	0.0354 (9)	0.0011 (5)	0.0024 (6)	0.0027 (6)
N6	0.0471 (9)	0.0417 (8)	0.0204 (8)	−0.0003 (6)	0.0073 (6)	−0.0018 (7)
N7	0.0266 (7)	0.0222 (6)	0.0185 (7)	−0.0007 (5)	0.0024 (5)	−0.0007 (6)
N8	0.0214 (6)	0.0250 (6)	0.0170 (7)	−0.0028 (5)	0.0029 (5)	0.0000 (5)
N9	0.0299 (7)	0.0342 (7)	0.0182 (7)	−0.0078 (5)	0.0045 (5)	0.0019 (6)
N10	0.0275 (7)	0.0257 (7)	0.0256 (8)	−0.0025 (5)	0.0004 (6)	−0.0010 (6)
C1	0.0243 (8)	0.0389 (9)	0.0330 (10)	0.0029 (7)	0.0007 (7)	−0.0018 (8)
C2	0.0274 (8)	0.0322 (9)	0.0431 (12)	−0.0046 (6)	0.0024 (7)	−0.0014 (8)
C3	0.0310 (9)	0.0248 (8)	0.0392 (11)	0.0023 (6)	0.0031 (7)	0.0020 (8)
C4	0.0252 (8)	0.0270 (8)	0.0236 (9)	0.0028 (6)	0.0037 (6)	−0.0019 (7)
C5	0.0291 (8)	0.0256 (8)	0.0352 (10)	−0.0008 (6)	0.0002 (7)	−0.0008 (7)
C6	0.0260 (8)	0.0264 (8)	0.0278 (9)	0.0006 (6)	0.0031 (7)	−0.0001 (7)
C7	0.0257 (8)	0.0265 (8)	0.0313 (10)	0.0039 (6)	0.0080 (7)	0.0031 (7)
C8	0.0246 (8)	0.0332 (8)	0.0267 (9)	0.0011 (6)	0.0049 (7)	0.0029 (7)
C9	0.0271 (8)	0.0246 (8)	0.0299 (10)	0.0011 (6)	0.0022 (7)	0.0017 (7)
C10	0.0313 (9)	0.0361 (9)	0.0482 (12)	−0.0030 (7)	−0.0046 (8)	0.0022 (9)
C11	0.0496 (11)	0.0346 (9)	0.0233 (10)	0.0021 (8)	−0.0032 (8)	−0.0025 (8)
C12	0.0341 (9)	0.0313 (9)	0.0344 (11)	−0.0002 (7)	−0.0015 (7)	−0.0027 (8)
C13	0.0357 (9)	0.0231 (8)	0.0270 (9)	−0.0011 (6)	0.0078 (7)	−0.0009 (7)
C14	0.0320 (8)	0.0206 (7)	0.0219 (9)	−0.0001 (6)	0.0052 (6)	−0.0004 (7)
C15	0.0358 (9)	0.0349 (9)	0.0249 (9)	−0.0010 (7)	0.0079 (7)	−0.0005 (8)
C16	0.0270 (8)	0.0268 (8)	0.0221 (9)	−0.0006 (6)	0.0064 (6)	−0.0003 (7)
C17	0.0268 (8)	0.0190 (7)	0.0207 (8)	−0.0017 (6)	0.0067 (6)	−0.0010 (6)
C18	0.0257 (8)	0.0194 (7)	0.0267 (9)	0.0016 (6)	0.0023 (7)	−0.0004 (7)
C19	0.0223 (7)	0.0240 (7)	0.0243 (9)	−0.0006 (5)	0.0059 (6)	0.0024 (7)
C20	0.0268 (8)	0.0328 (8)	0.0367 (10)	−0.0015 (6)	0.0021 (7)	0.0004 (8)

### Geometric parameters (Å, º)

**Table:**

O1—C7	1.2202 (18)	C4—C5	1.385 (2)
O2—C17	1.2263 (16)	C4—C6	1.461 (2)
N1—C1	1.331 (2)	C5—H5	0.9500
N1—C5	1.3352 (19)	C6—H6	0.9500
N2—C6	1.2797 (19)	C8—C10	1.491 (2)
N2—N3	1.3719 (17)	C8—C9	1.492 (2)
N3—C7	1.3807 (18)	C9—H9A	0.9900
N3—C9	1.4534 (18)	C9—H9B	0.9900
N4—C7	1.3594 (19)	C10—H10A	0.9800
N4—N5	1.3832 (17)	C10—H10B	0.9800
N4—H4	0.8800	C10—H10C	0.9800
N5—C8	1.2701 (19)	C11—C12	1.383 (2)
N6—C11	1.332 (2)	C11—H11	0.9500
N6—C15	1.335 (2)	C12—C13	1.380 (2)
N7—C16	1.281 (2)	C12—H12	0.9500
N7—N8	1.3730 (17)	C13—C14	1.388 (2)
N8—C17	1.3795 (19)	C13—H13	0.9500
N8—C19	1.4536 (17)	C14—C15	1.394 (2)
N9—C17	1.3564 (19)	C14—C16	1.461 (2)
N9—N10	1.3867 (17)	C15—H15	0.9500
N9—H9	0.8800	C16—H16	0.9500
N10—C18	1.274 (2)	C18—C20	1.491 (2)
C1—C2	1.377 (2)	C18—C19	1.492 (2)
C1—H1	0.9500	C19—H19A	0.9900
C2—C3	1.379 (2)	C19—H19B	0.9900
C2—H2	0.9500	C20—H20A	0.9800
C3—C4	1.387 (2)	C20—H20B	0.9800
C3—H3	0.9500	C20—H20C	0.9800
			
C1—N1—C5	116.61 (13)	N3—C9—H9B	109.2
C6—N2—N3	117.97 (12)	C8—C9—H9B	109.2
N2—N3—C7	115.55 (12)	H9A—C9—H9B	107.9
N2—N3—C9	120.66 (11)	C8—C10—H10A	109.5
C7—N3—C9	123.66 (12)	C8—C10—H10B	109.5
C7—N4—N5	127.42 (12)	H10A—C10—H10B	109.5
C7—N4—H4	116.3	C8—C10—H10C	109.5
N5—N4—H4	116.3	H10A—C10—H10C	109.5
C8—N5—N4	117.31 (13)	H10B—C10—H10C	109.5
C11—N6—C15	117.07 (15)	N6—C11—C12	123.42 (16)
C16—N7—N8	117.67 (12)	N6—C11—H11	118.3
N7—N8—C17	115.60 (11)	C12—C11—H11	118.3
N7—N8—C19	121.02 (12)	C13—C12—C11	118.97 (16)
C17—N8—C19	123.36 (12)	C13—C12—H12	120.5
C17—N9—N10	127.41 (13)	C11—C12—H12	120.5
C17—N9—H9	116.3	C12—C13—C14	118.91 (16)
N10—N9—H9	116.3	C12—C13—H13	120.5
C18—N10—N9	116.63 (13)	C14—C13—H13	120.5
N1—C1—C2	123.36 (14)	C13—C14—C15	117.63 (15)
N1—C1—H1	118.3	C13—C14—C16	124.17 (15)
C2—C1—H1	118.3	C15—C14—C16	118.20 (14)
C1—C2—C3	119.24 (15)	N6—C15—C14	123.96 (15)
C1—C2—H2	120.4	N6—C15—H15	118.0
C3—C2—H2	120.4	C14—C15—H15	118.0
C2—C3—C4	118.79 (14)	N7—C16—C14	120.11 (14)
C2—C3—H3	120.6	N7—C16—H16	119.9
C4—C3—H3	120.6	C14—C16—H16	119.9
C5—C4—C3	117.30 (14)	O2—C17—N9	121.57 (14)
C5—C4—C6	119.41 (13)	O2—C17—N8	123.36 (14)
C3—C4—C6	123.28 (13)	N9—C17—N8	115.07 (12)
N1—C5—C4	124.65 (14)	N10—C18—C20	118.78 (14)
N1—C5—H5	117.7	N10—C18—C19	125.12 (13)
C4—C5—H5	117.7	C20—C18—C19	116.10 (14)
N2—C6—C4	119.22 (14)	N8—C19—C18	112.06 (12)
N2—C6—H6	120.4	N8—C19—H19A	109.2
C4—C6—H6	120.4	C18—C19—H19A	109.2
O1—C7—N4	121.57 (14)	N8—C19—H19B	109.2
O1—C7—N3	123.81 (14)	C18—C19—H19B	109.2
N4—C7—N3	114.60 (13)	H19A—C19—H19B	107.9
N5—C8—C10	119.06 (14)	C18—C20—H20A	109.5
N5—C8—C9	124.62 (14)	C18—C20—H20B	109.5
C10—C8—C9	116.31 (13)	H20A—C20—H20B	109.5
N3—C9—C8	112.26 (12)	C18—C20—H20C	109.5
N3—C9—H9A	109.2	H20A—C20—H20C	109.5
C8—C9—H9A	109.2	H20B—C20—H20C	109.5
			
C6—N2—N3—C7	177.92 (15)	C7—N3—C9—C8	1.3 (2)
C6—N2—N3—C9	−6.1 (2)	N5—C8—C9—N3	−1.1 (2)
C7—N4—N5—C8	4.1 (2)	C10—C8—C9—N3	178.12 (14)
C16—N7—N8—C17	172.24 (12)	C15—N6—C11—C12	0.4 (2)
C16—N7—N8—C19	−6.07 (19)	N6—C11—C12—C13	1.4 (3)
C17—N9—N10—C18	4.7 (2)	C11—C12—C13—C14	−1.7 (2)
C5—N1—C1—C2	−1.8 (3)	C12—C13—C14—C15	0.2 (2)
N1—C1—C2—C3	1.3 (3)	C12—C13—C14—C16	179.96 (14)
C1—C2—C3—C4	0.8 (3)	C11—N6—C15—C14	−2.0 (2)
C2—C3—C4—C5	−2.1 (2)	C13—C14—C15—N6	1.7 (2)
C2—C3—C4—C6	178.24 (16)	C16—C14—C15—N6	−178.07 (14)
C1—N1—C5—C4	0.3 (3)	N8—N7—C16—C14	179.00 (12)
C3—C4—C5—N1	1.7 (3)	C13—C14—C16—N7	−8.9 (2)
C6—C4—C5—N1	−178.69 (16)	C15—C14—C16—N7	170.84 (14)
N3—N2—C6—C4	179.19 (14)	N10—N9—C17—O2	174.79 (13)
C5—C4—C6—N2	174.00 (15)	N10—N9—C17—N8	−5.0 (2)
C3—C4—C6—N2	−6.4 (3)	N7—N8—C17—O2	1.8 (2)
N5—N4—C7—O1	177.59 (16)	C19—N8—C17—O2	−179.92 (13)
N5—N4—C7—N3	−3.9 (2)	N7—N8—C17—N9	−178.42 (11)
N2—N3—C7—O1	−4.7 (2)	C19—N8—C17—N9	−0.15 (19)
C9—N3—C7—O1	179.44 (15)	N9—N10—C18—C20	−178.69 (13)
N2—N3—C7—N4	176.77 (13)	N9—N10—C18—C19	0.8 (2)
C9—N3—C7—N4	1.0 (2)	N7—N8—C19—C18	−177.33 (11)
N4—N5—C8—C10	179.51 (15)	C17—N8—C19—C18	4.49 (19)
N4—N5—C8—C9	−1.3 (2)	N10—C18—C19—N8	−5.0 (2)
N2—N3—C9—C8	−174.34 (14)	C20—C18—C19—N8	174.58 (12)

### Hydrogen-bond geometry (Å, º)

**Table:**

*D*—H···*A*	*D*—H	H···*A*	*D*···*A*	*D*—H···*A*
N4—H4···O2^i^	0.88	2.32	2.9545 (17)	129
N4—H4···N7^i^	0.88	2.43	3.2346 (17)	152
N9—H9···N6^ii^	0.88	2.04	2.882 (2)	159
C19—H19*A*···N1^iii^	0.99	2.57	3.0852 (19)	112
C19—H19*A*···O1^iv^	0.99	2.60	3.5596 (19)	164
C20—H20*C*···O1^v^	0.98	2.53	3.3906 (19)	147

Symmetry codes: (i) *x*−1/2, −*y*+1/2, *z*+1/2; (ii) *x*, *y*, *z*−1; (iii) *x*−1, *y*, *z*−1; (iv) *x*−1/2, −*y*+1/2, *z*−1/2; (v) −*x*+1/2, *y*−1/2, −*z*+1/2.
