# Supplementary figures and images for: Crystal structure of pymetrozine
Source: Acta Crystallogr E Crystallogr Commun. 2015 Jun 10;71(Pt 7):o461–2. doi: 10.1107/S2056989015010804 (PMC4518907; doi:10.1107/S2056989015010804)

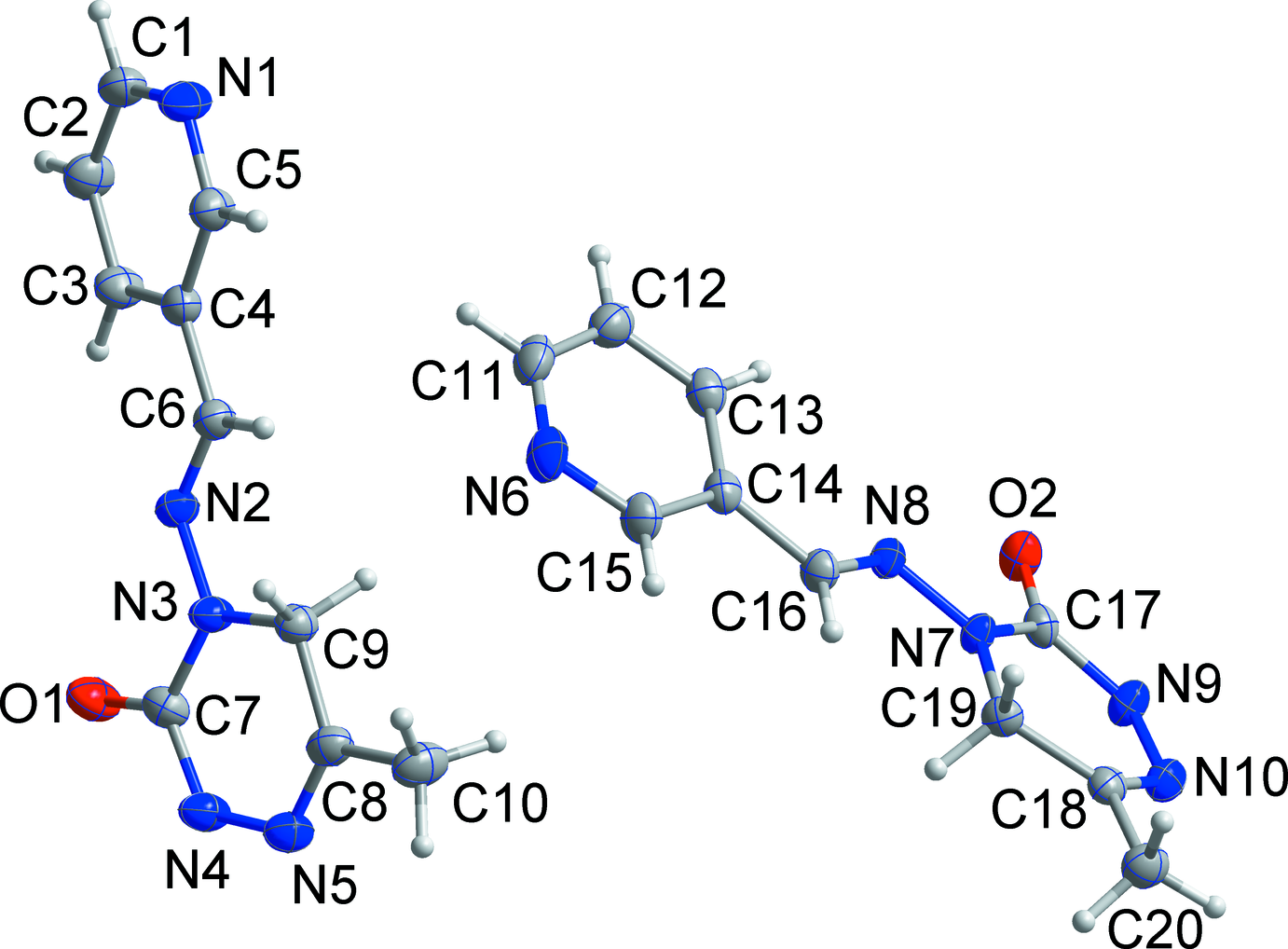

Supplement: Supplementary file 4 [file e-71-0o461-fig1.tif]

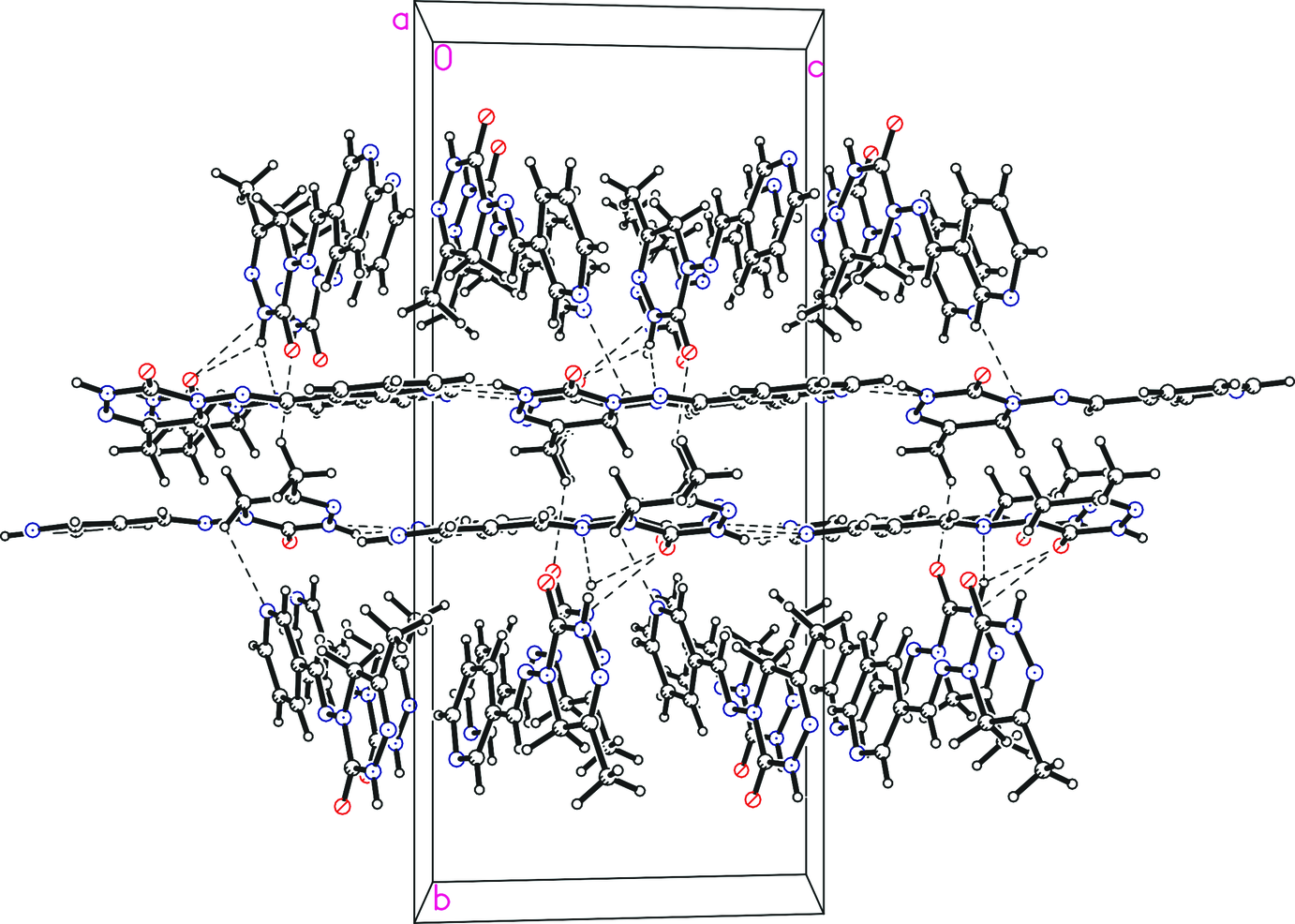

Supplement: Supplementary file 5 [file e-71-0o461-fig2.tif]
